# Human vascular endothelial cells express epithelial growth factor in response to infection by *Bartonella bacilliformis*

**DOI:** 10.1371/journal.pntd.0008236

**Published:** 2020-04-17

**Authors:** Linda D. Hicks, Michael F. Minnick

**Affiliations:** Program in Cellular, Molecular & Microbial Biology, Division of Biological Sciences, University of Montana, Missoula, Montana, United States of America; Yale University School of Medicine, UNITED STATES

## Abstract

*Bartonella* are Gram-negative bacterial pathogens that trigger pathological angiogenesis during infection of humans. *Bartonella bacilliformis* (*Bb*) is a neglected tropical agent endemic to South America, where it causes Carrión’s disease. Little is known about *Bb*’s virulence determinants or how the pathogen elicits hyperproliferation of the vasculature, culminating in Peruvian warts (verruga peruana) of the skin. In this study, we determined that active infection of human umbilical vein endothelial cells (HUVECs) by live *Bb* induced host cell secretion of epidermal growth factor (EGF) using ELISA. Killed bacteria or lysates of various *Bb* strains did not cause EGF production, suggesting that an active infection was necessary for the response. *Bb* also caused hyperproliferation of infected HUVECs, and the mitogenic response could be inhibited by the EGF-receptor (EGFR) inhibitor, AG1478. *Bb* strains engineered to overexpress recombinant GroEL, evoked greater EGF production and hyperproliferation of HUVECs compared to control strains. Conditioned (spent) media from cultured HUVECs that had been previously infected by *Bb* were found to be mitogenic for naïve HUVECs, and the response could be inhibited by EGFR blocking with AG1478. *Bb* cells and cell lysates stimulated HUVEC migration and capillary-like tube formation in transmigration and Matrigel assays, respectively. To our knowledge, this is the first demonstration of EGF production by *Bb*-infected endothelial cells; an association that could contribute to hyperproliferation of the vascular bed during bartonellosis.

## Author summary

*Bartonella* are bacteria that infect the circulatory system and, unlike other bacteria, cause blood vessels to grow uncontrollably in the skin, spleen and liver of humans. In many respects, the process resembles the aberrant blood vessel formation that occurs during tumor formation. This study found that when *Bartonella bacilliformis* (*Bb*) infects vascular endothelial cells (VECs) that line the circulatory system, it causes them to overproduce a protein called epidermal growth factor (EGF) which, in turn, causes the cells to multiply more rapidly than usual. We also found that VECs migrate towards the bacterium and form capillary-like tubes; processes that occur during an actual infection. This cause-effect has not been previously reported, and it may help to explain the pathology observed in humans infected by *Bb*.

## Introduction

*Bartonella* are arthropod-transmitted, Gram-negative bacteria that parasitize the circulatory system of mammals. *Bartonella bacilliformis* (*Bb*), *Bartonella quintana* (*Bq*) and *Bartonella henselae* (*Bh*); etiological agents of Carrión’s disease, trench fever, and cat-scratch disease, respectively, are the major pathogenic species for humans. Bartonelloses are manifested by diverse symptoms and syndromes, including chronic asymptomatic bacteremia, infectious endocarditis, bacillary angiomatosis, bacillary peliosis and hemolytic anemia. These disorders arise primarily through parasitism of two host cell types. First, bartonellae are hemotrophic pathogens that infect erythrocytes, presumably to fulfill their extraordinary requirement for heme [1]. Hemotrophy is an unusual parasitic strategy for bacteria, and likely contributes to the severe hemolytic anemia observed in the acute (hematic) phase of Carrión’s disease and the persistent bacteremia common to all types of bartonelloses [1]. Second, *Bartonella* infects vascular endothelial cells (VECs) and evokes pathological angiogenesis in the skin, spleen and liver of humans, ultimately leading to bacillary angiomatosis (*Bq* or *Bh*), verruga peruana (*Bb*), and bacillary peliosis (*Bh*) [1].

Pathological angiogenesis induced by *Bh* arises directly from infected VECs and indirectly through the influence of effector cells in the vicinity of the infection. For example, VECs infected by *Bh* have been shown to secrete the angiogenic chemokine IL-8, upregulate expression of the IL-8 receptor (CXCR2), and display elevated Bcl-2: Bax ratios that delay apoptosis [2]. *Bh*-infected VECs also produce monocyte-macrophage chemoattractant protein-1 (MCP-1) which recruits various cell types such as monocytes [3]; a source of pro-angiogenic vascular endothelial cell growth factor (VEGF) [3, 4]. Moreover, *Bh-*infected THP-1 macrophages or EA.hy 926 cells (a VEC hybrid line) were found to produce VEGF as well as IL-1β, a potentiator of VEGF [4, 5]. Heavily-infected human umbilical vein endothelial cells (HUVECs) have also been shown to upregulate expression of VEGF, IL-8 and hypoxia-inducible factor-I (HIF-1); a key transcriptional factor for angiogenesis [6]. Finally, research has demonstrated the importance of the *Bh* VirB/VirD4 type IV secretion system (T4SS), its translocated substrates [i.e., *Bartonella* effector proteins (Beps)] and direct host cell contact via the *Bartonella* adhesin A (BadA) protein, in evoking a pro-inflammatory response and inhibiting apoptosis to ultimately foster neovascularization [7, 8, 9, 10].

While the molecular basis for pathological angiogenesis during a *Bh* infection has been investigated in considerable detail, it is relatively understudied in *Bb*. This is unfortunate, as *Bb* is the most virulent *Bartonella* species and employs a set of virulence determinants that are distinct from those of *Bh* and *Bq* [1, 11]. For example, *Bb* lacks the VirB/VirD4 T4SS and effector substrates employed by *Bh* and *Bq*; thus, they are obviously not involved in the pathological angiogenesis induced by this bacterium. The earliest report on *Bb*-induced angiogenesis showed that the soluble fraction of the *Bb* cell contained a heat-sensitive factor(s) that was significantly and specifically mitogenic for cultured HUVECs [12]. In addition, the soluble fraction was able to evoke VEC invasion into subcutaneous sponge implants in a Sprague-Dawley rat model [12]. Subsequent work by our lab set out to purify the responsible factor(s) from the *Bb* cell lysate and identified GroEL as a participant in HUVEC mitogenicity [13]. Results showed that *in vitro* proliferation of HUVECs correlated with levels of GroEL present in the *Bb* cell lysate, and anti-*Bb* GroEL antibodies significantly decreased the mitogenic activity [13]. Interestingly, a *Bh* cell lysate showed significantly less mitogenicity for HUVECs as compared to *Bb* cell lysates, underscoring the differences between *Bb*- and *Bh*-induced angiogenesis.

The current study was undertaken to address the hypothesis that *Bb* infection of HUVECs induces the expression of potentially angiogenic protein factors, and that GroEL might play a role in the process, based upon our earlier work [13]. The results of the study showed that a *Bb* infection of HUVECs induced expression of epidermal growth factor (EGF) which, in turn, significantly enhanced cell proliferation *in vitro*. To our knowledge, this is the first description of EGF production by VECs in direct response to a *Bartonella* infection and discussion of the growth factor’s potential role in pathological angiogenesis.

## Materials and methods

### Bacterial culture and cell preparations

*Bb* strains were cultivated on Bacto heart infusion agar (Becton Dickinson; Franklin Lakes, NJ) containing 4% defibrinated sheep blood and 2% sheep serum (HIBB plates) by volume. *Bb* cultures were routinely grown for 4 d at 30^°^C in a water-saturated atmosphere under ambient CO_2_ levels. *Escherichia coli* (TOP10F′) was grown for 16 h at 37°C in lysogeny broth (LB), with shaking or on LB agar plates. Antibiotic supplements were added to media as required, including kanamycin (25 μg/ml) or ampicillin (100 μg/ml). A complete list of bacterial strains used in the study is provided in **S1 Table**.

Whole-cell lysates from the *Bb* strains listed in **S1 Table** were prepared as previously described [13] with minor modifications. Briefly, 4-d-old cultures of *Bb* were harvested from two HIBB plates into ice-cold phosphate-buffered saline (PBS; pH 7.4). The mixture was transferred to a sterile, 2-ml screw-cap microcentrifuge tube containing ~500 μl of 0.1-mm diameter glass beads. Bacterial cells were lysed for 5 min with a Disruptor Genie (Scientific Industries; Bohemia, NY). Cellular debris was subsequently removed by centrifugation at 16,000 x g for 5 min at 4^°^C. The supernatant was transferred to a sterile microcentrifuge tube, assayed for protein content using a Pierce BCA Protein Assay kit as instructed (ThermoFisher; Waltham, MA) and stored at -20^°^C until used. Formaldehyde-fixed (killed) *Bb* strains were prepared by harvesting and washing 4-d-old cultures in Endothelial Cell Growth Basal Medium-Plus (Lonza; Greenwood, SC) followed by fixing in 1% (v/v) formaldehyde (ThermoFisher) for 24 h at 4^°^C. Fixed bacteria were washed with, and resuspended in, EBM+ then stored at -20^°^C until used.

### Genetic manipulation

Genomic DNA was purified using a DNeasy kit as instructed (Qiagen; Germantown, MD). A Q5 Site-Directed Mutagenesis kit was employed to generate deletions in successive portions of the *Bb groESL* operon cloned in pGRO1 (see **S1 Fig**), following manufacturer’s instructions (New England Biolabs; Ipswich, MA). Primers used in deletion mutagenesis are listed in **S2 Table**. *Bb* (strain JB584) was transformed by electroporation, as previously described [14], using ~0.1–4 μg purified plasmid DNA. DNA was cleaned prior to electroporation with Ultra-0.5 ml Amicon filters (Millipore Sigma; Burlington, MA) and stored in nuclease-free water until used. Plasmid content was verified before and after transformation by Sanger automated sequencing with pbbrSEQ_F and pbbrSEQ_R primers (**S2 Table**).

### HUVEC cultivation and infections

Pooled, primary HUVECs (PCS-100-013) were obtained from the American Type Culture Collection (ATCC; Manassas, VA). For routine maintenance, HUVECs were cultured essentially as previously described [13], but in Endothelial Cell Growth Basal Medium-Plus (Lonza) supplemented with an Endothelial Cell Growth Kit-BBE, as instructed by the supplier (ATCC). HUVECs employed in the study were restricted to passages two through five. Prior to the start of infections, HUVEC growth medium was replaced with Endothelial Cell Growth Basal Media-Plus (EBM+) containing 10 mM L-glutamine plus 0.5% fetal bovine serum (Rocky Mountain Biologicals; Missoula, MT) to slow growth. Endothelial cells were infected at a MOI of 500 bacteria per mammalian cell; bacteria from 4-d-old plates were harvested and washed using EBM+ prior to infections. Bacterial cell numbers were quantified using a LIVE/DEAD BacLight kit (ThermoFisher; L7007). Unless otherwise indicated, HUVECs were infected for 24 h at 37^°^C and 5% CO_2_ following serum starvation.

### Growth assays

For cell growth experiments, serum-starved HUVECs were harvested and used to seed a 96-well plate at a density of 1000 cells per well using EBM+ 20% fetal bovine serum (FBS). Wells were brought to a final volume of 100 μl with cell culture medium after the addition of prepared *Bb* lysate, PBS, VEGF (PHC9394) (Gibco; Gaithersburg, MD) at 10 ng/ml, prepared conditioned media (CM) or infected with the indicated strains of *Bb* (MO1 = 500). CM were prepared by removing cell culture media from each well following infection and centrifuged at 4^°^C to remove any cellular debris; media were then passed through a 0.22-μm syringe filter to sterilize, and each infection was done individually with a new filter and kept on ice. Once sterilized, CM was aliquoted to limit freeze/thaw cycles and stored at -80^°^C until used. HUVEC numbers were measured indirectly using a phosphatase assay for viable cells following a 96-h growth period at 37^°^C, 5% CO_2_ [15]. For growth assays involving an epidermal growth factor receptor (EGFR) inhibitor; AG1478 (Sigma-Aldrich; St. Louis, MO) was added to a final concentration of 10 μM to the wells immediately prior to infection with *Bb* strains.

### HUVEC migration and tube-formation assays

HUVEC migration was analyzed using a transmigration assay as described in the Endothelial Cell Migration and Invasion Assay application note from PromoCell; Heidelberg, Germany [16]. Briefly, low passage HUVECs were serum-starved for 16 h prior to the start of the assay in EBM+ 10 mM L-glutamine. Cells (1 x 10^5^) were added to the upper chamber containing 200 μl of base media without FBS, while 600 μl of base media with 2% FBS along with lysates (1 μg / ml protein), live *Bb* strains or untreated controls were added to the lower chamber. HUVECs that migrated through the membrane after 20 h were fixed in methanol and stained with 0.2% crystal violet. To determine the number of migrated cells, the mean value was calculated by counting the number of cells in five random fields (at ~100–200 cells per field) under an inverted light microscope.

Tube formation by HUVECs, in response to exposure to *Bb* cell lysates or live *Bb* strains, was examined by the methods of Arnaoutova and Kleinman [17]. Briefly, 50 μl / cm^2^ of Matrigel Basement Membrane Matrix Growth Factor Reduced (354230) (Corning; Corning, NY) was added to the surface of a tissue culture plate and allowed to solidify for 30 min at 37^°^C. HUVECs were diluted in either EBM or EBM+ 2% FBS to achieve 9.0x10^4^ cells per well and overlaid onto the surface of solidified Matrigel. *Bb* cell lysates, VEGF (10 ng/ml) or live *Bb* strains (MOI = 500) were added to indicated wells and incubated for 6 h to allow for development of tubes. HUVECs were subsequently stained for 30 min with Calcein AM at 2 μM as instructed by the manufacturer (Corning; 354216). Five random images from each well were captured at 40X magnification under an inverted light microscope and images quantified using ImageJ Angiogenesis Analyzer plugin focusing on formation of nodes and branches [18].

### Angiogenic factor arrays and ELISA

Angiogenesis arrays were used to analyze differential expression of twenty human cytokines by HUVECs in response to a *Bb* infection. HUVECs were infected for 24 h with various *Bb* strains (MOI = 500). Culture supernatants were subsequently collected and analyzed by a Human Angiogenesis Antibody Array C1 as instructed by the manufacturer (RayBiotech; Peachtree Corners, GA). Results of the array were interpreted by the Human Angiogenesis Analysis Tool (RayBiotech). A RayBio Human EGF ELISA kit was subsequently used to assay changes in EGF concentrations in the HUVEC culture medium in response to *Bb* lysates, formaldehyde-fixed *Bb* or *Bb*-infection at 24 h post-infection or post-treatment, following the manufacturer’s instructions (RayBiotech).

### Statistics and graphics

Statistical analyses were performed using Prism (8.2.0) software (GraphPad; San Diego, CA). Statistical significance was determined by a Student’s *t-*test, where a *P-*value of < 0.05 was considered significant. Graphics were done using Excel and Powerpoint software (Microsoft; Redmond, WA).

## Results

**Fig 1:**
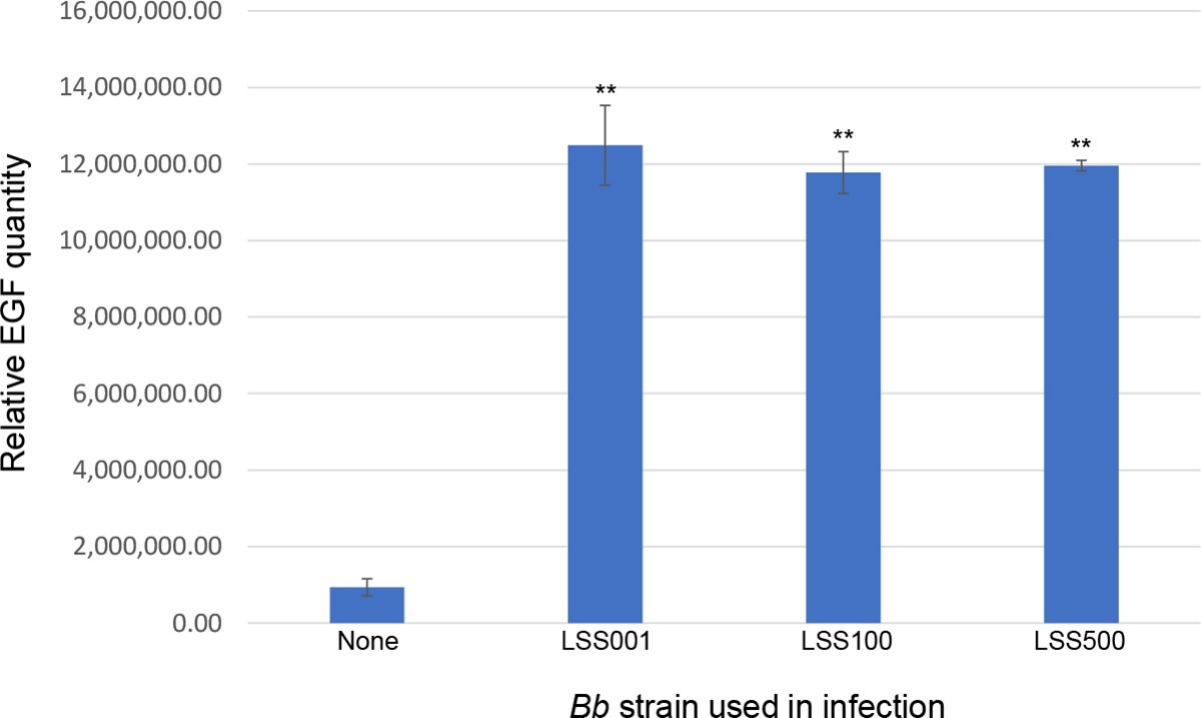
Detection of EGF produced by *Bb*-infected HUVECs. Results of an angiogenic factor array (RayBiotech) are shown with relative EGF levels in the HUVEC culture medium following a 24-h infection by three different *Bb* strains as indicated. Uninfected HUVECs were included as a negative control. Values represent the means of two independent determinations ± SEM. (** *P* < 0.01 relative to uninfected controls).

During work exploring the molecular basis for *Bb*-induced pathological angiogenesis, we determined that a 24-h infection by the bacterium resulted in a significant increase in expression and secretion of epidermal growth factor (EGF) into the culture medium as compared to uninfected control cells (**Fig 1**). In addition, the magnitude of increase in EGF production, relative to controls, was similar between the three *Bb* strains employed, including LSS001, LSS100 and LSS500. (Please see **S1 Fig and S1 Table** for details on *Bb* strains).

**Fig 2:**
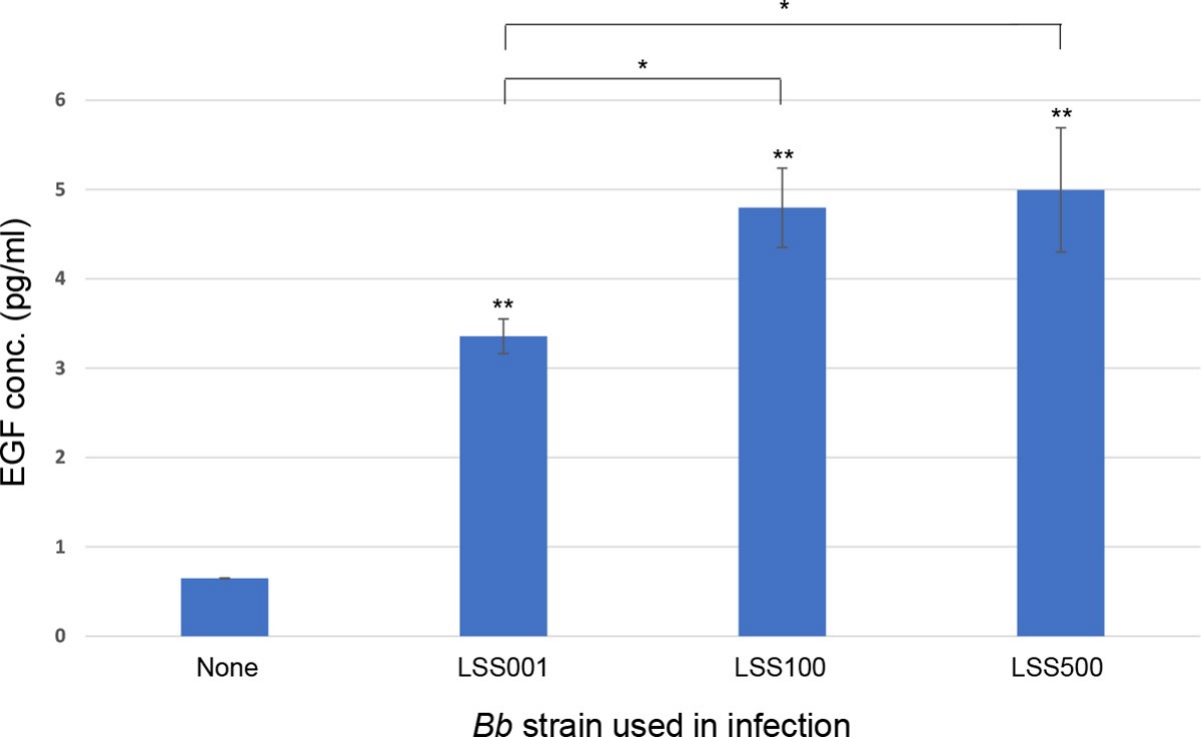
EGF concentrations in the HUVEC culture medium following a *Bb* infection. ELISA analysis of EGF concentrations (pg/ml) in HUVEC culture media following a 24-h infection by three different *Bb* strains is shown. Values represent the average of three independent determinations done in duplicate ± SEM. (* *P* < 0.05 relative to indicated pair; ** *P* < 0.01 relative to uninfected controls).

To quantify the EGF present in the growth medium following infection, a human EGF ELISA (RayBiotech) was employed. Results of the ELISA revealed significant increases in EGF production (P <0.01) in response to infections by all three *Bb* strains compared to mock-infected controls, with concentrations ranging from ~3 pg EGF / ml (strain LSS001) to 5 pg EGF / ml (strains LSS100 and LSS500) (**Fig 2**). In addition, the quantity of EGF released in response to infection by strains LSS100 and LSS500 (overexpressing rGroEL) was significantly higher than that elicited by strain LSS001 (harboring the corresponding vector) (P < 0.05) (**Fig 2**).

**Fig 3:**
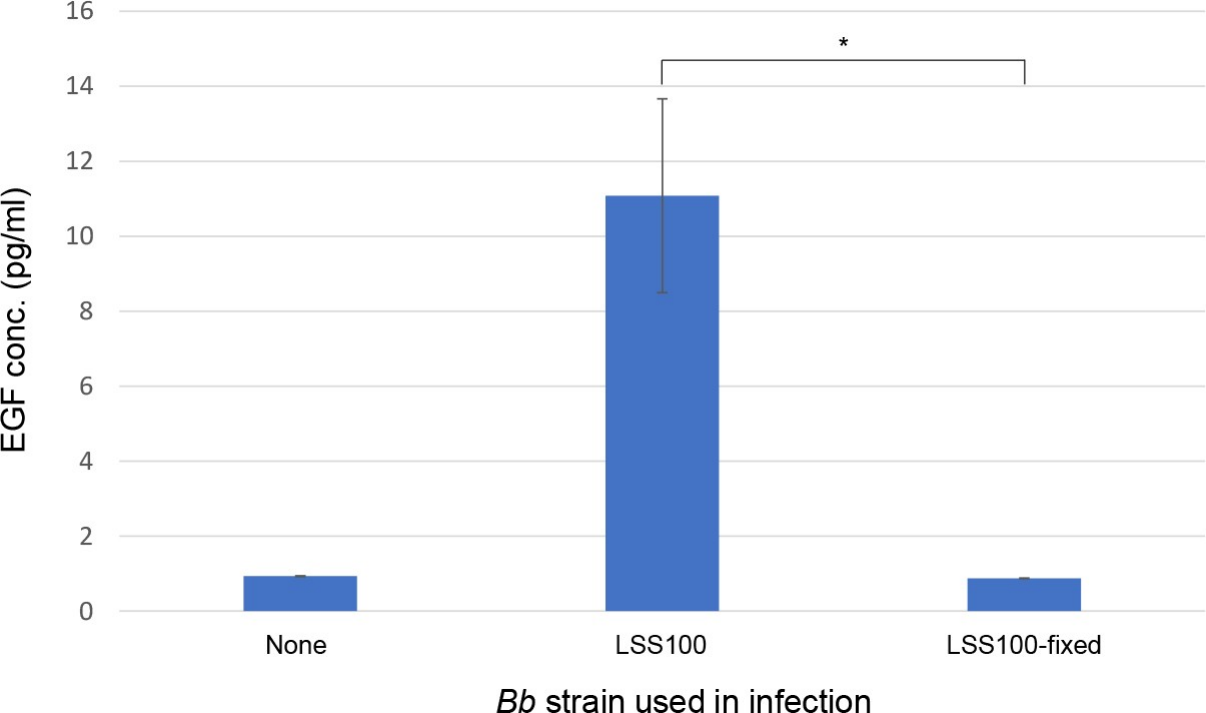
EGF production by HUVECs requires infection by viable *Bb*. ELISA determination of EGF concentrations (pg/ml) in HUVEC culture medium following a 24-h infection with viable or non-viable (formaldehyde-fixed) cells of *Bb* strain LSS100. Uninfected HUVECs were included as a negative control. Values represent the means of three independent determinations involving *Bb* and two independent determinations for uninfected HUVECs ± SEM. (* *P* < 0.05).

Secretion of EGF by HUVECs in response to infection by *Bb* made us curious whether the reaction was due to an active infection or simply arose through bacterial interactions with the host cell. To address this, we conducted parallel experiments in which HUVECs were challenged for 24 h with live or non-viable *Bb* LSS100, then assayed the medium for secreted EGF by ELISA. Results of these experiments showed a significant decrease in EGF production in response to non-viable versus live *Bb* LSS100 (*P* < 0.05) (**Fig 3**). In fact, EGF production levels were similar between uninfected HUVECs and what was observed following challenge with non-viable bacteria (**Fig 3**). Similarly, treatment of HUVECs for 24 h with cell lysates obtained from three *Bb* strains did not significantly alter the amount of EGF released to the media relative to untreated cultures (all ~0.65 pg/ml; **S2 Fig**).

**Fig 4:**
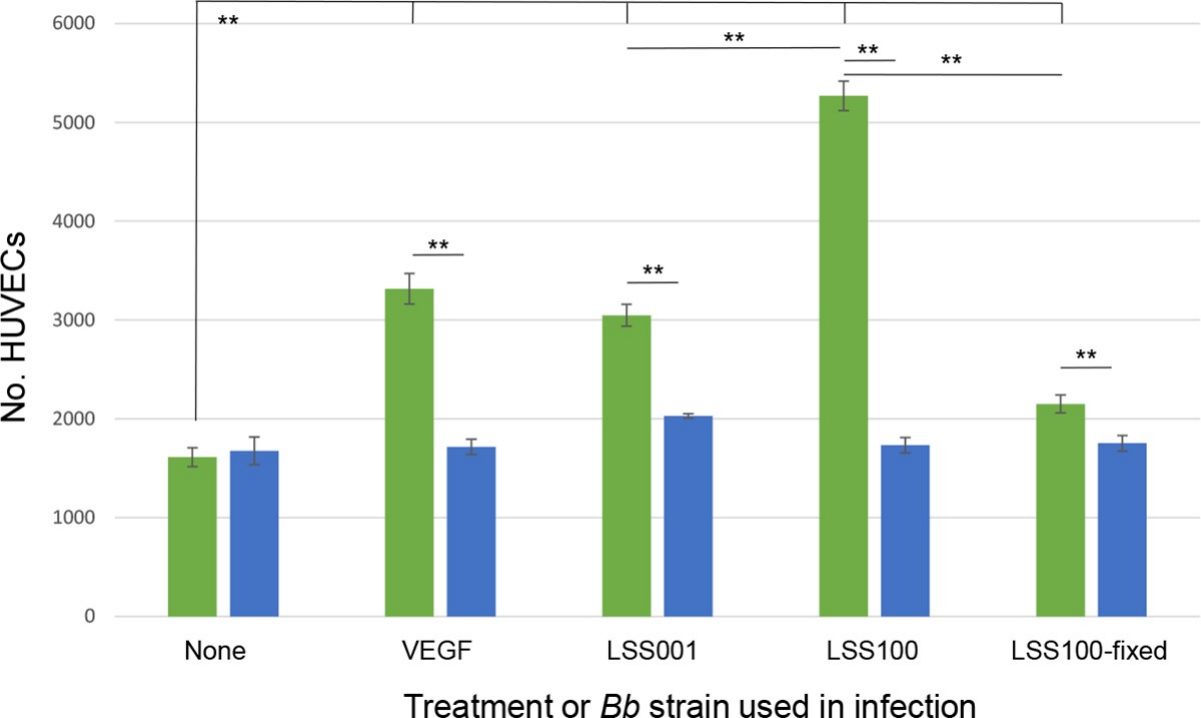
Blocking EGFR inhibits HUVEC proliferation during *Bb* infection. Results of HUVEC proliferation assays following a 96-h infection by two different *Bb* strains in the absence (green bars) or presence of AG1478 (10 μM; blue bars) to block EGFR. Controls included untreated HUVECs, treatment with VEGF (10 ng/ml), and mock infection with non-viable (formaldehyde-fixed) bacteria. This experiment was conducted three times independently with indistinguishable results. Results of one experiment are shown, and values represent the means of six technical replicates ± SEM. (** *P* < 0.01).

Since EGF could conceivably play a role in the hyperproliferation of VECs during bartonellosis of humans, we examined HUVEC proliferation in response to a 96-h infection by two live *Bb* strains (LSS001 and LSS100) and one non-viable *Bb* strain (LSS100-fixed) in the presence or absence of AG1478; a potent and specific EGFR-blocking agent [19]. These results showed that infections by any of the three *Bb* strains elicited a significant increase in HUVEC numbers relative to uninfected HUVECs (*P* < 0.01) (**Fig 4**). As expected, VEGF-treated positive-controls also showed a significant increase in cell numbers. The greatest relative HUVEC proliferation occurred in response to infection by *Bb* strain LSS100 (overexpressing GroEL), and it was significantly higher (*P* < 0.01) than what was produced by infection with *Bb* strain LSS001 (harboring the vector alone). As predicted, challenge by non-viable (fixed) *Bb* LSS100 resulted in HUVEC numbers that were similar to uninfected control cells. Finally, treatment with AG1478 significantly reduced HUVEC numbers (P < 0.01) compared to un-blocked infections or treatment with VEGF, and the resulting HUVEC numbers were similar to uninfected control cells. Only uninfected HUVECs did not respond to EGFR blocking by AG1478 (**Fig 4**).

**Fig 5:**
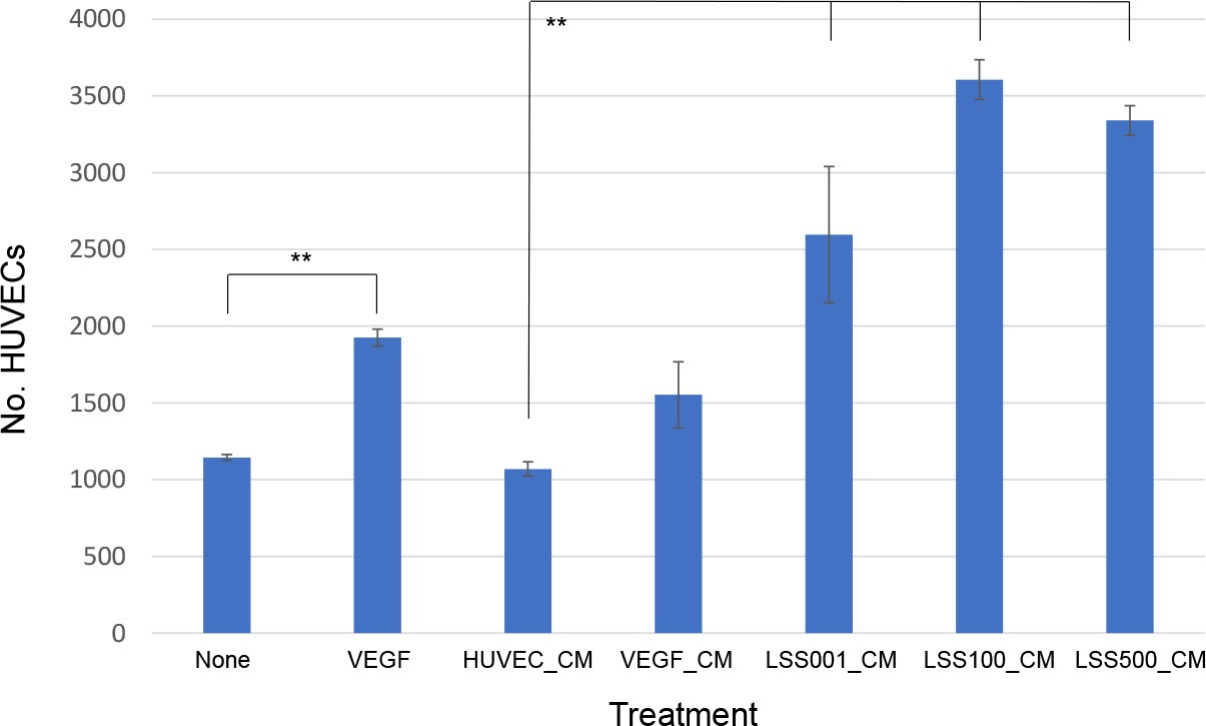
Conditioned media from *Bb*-infected HUVECs is mitogenic. HUVEC proliferation assay results following 96-h growth in conditioned media (CM) collected from HUVECs that had been previously infected for 24 h with one of three different *Bb* strains. Controls included 96-h growth of uninfected/untreated HUVECs, VEGF-treated HUVECs (10 ng/ml), or growth in CM from uninfected/untreated HUVECs or VEGF-treated HUVECs. Values represent the means of six independent determinations ± SEM. (** *P* < 0.01).

**Fig 6:**
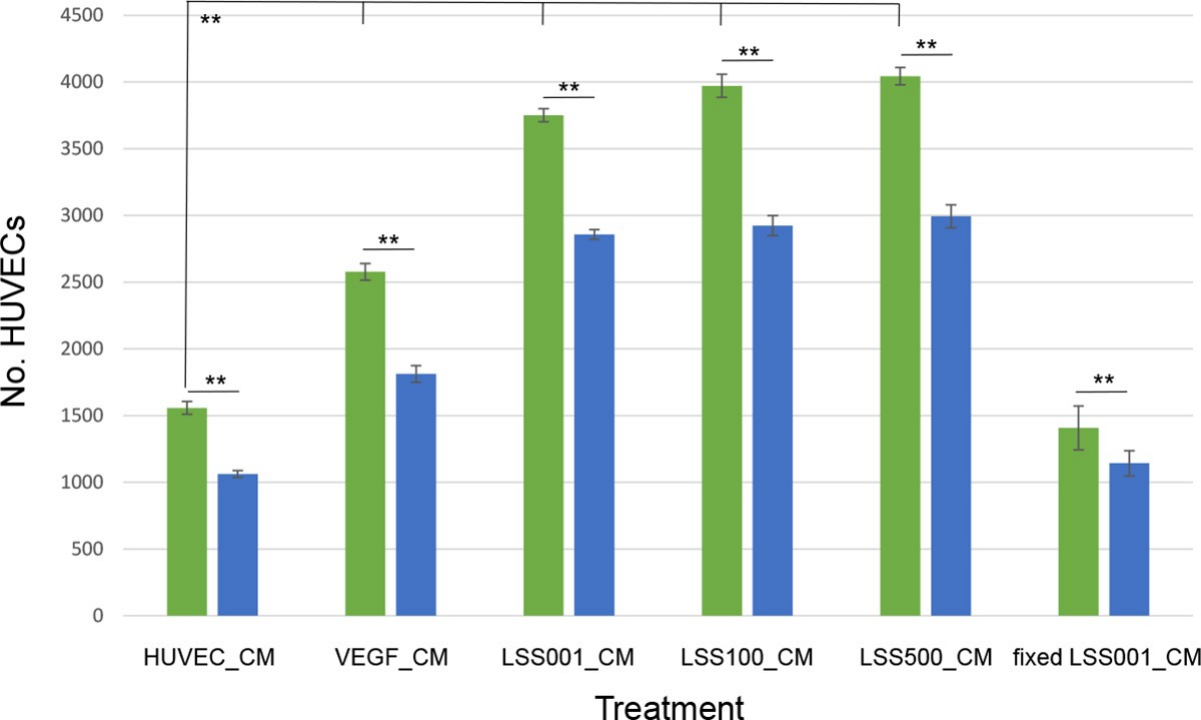
Blocking EGFR inhibits mitogenic activity of conditioned media. HUVEC proliferation assay results following 96-h growth in conditioned media (CM) from HUVECs that were infected for 24 h with the three indicated *Bb* strains or non-viable (formaldehyde-fixed) *Bb* LSS001. Controls included 96-h growth in CM obtained from 24-h uninfected/untreated HUVECs or VEGF-treated HUVECs. Parallel EGFR-blocking experiments (blue bars) were done by supplementing media with AG1478 (10 μM final concentration). (** *P* < 0.01).

Since the results indicated that EGF was secreted by *Bb*-infected HUVECs, we hypothesized that cell-free, CM from such cultures would be mitogenic if used to culture fresh, naive HUVECs. Indeed, results of this experiment showed that HUVEC numbers were significantly increased after 96 h of growth in CM from previous infections with three different *Bb* strains vs. a negative control of CM from untreated HUVECs (P < 0.01). In fact, HUVEC numbers were greater from the infection CMs than the CM collected from a VEGF positive-control (**Fig 5**). Although the greatest proliferation was observed in response to CM obtained from LSS100 / HUVEC co-cultures (nearly 3.5-fold higher than the negative control), proliferation results were not significantly different from those obtained with CM collected from LSS001- and LSS500- HUVEC co-cultures. To verify that the HUVEC proliferative response to the CM involved EGF, additional growth assays were done using AG1478. These results showed that blocking of EGFR by AG1478 significantly reduced HUVEC proliferation relative to unblocked cultures in the CM (**Fig 6**). However, unlike the *Bb* infection results shown in **Fig 4**, AG1478 did not uniformly block proliferation to levels approximating that of the negative control (i.e., HUVEC_CM). In addition, HUVEC proliferative responses to CM were similar regardless of *Bb* strain, and the response to CM collected from challenges with non-viable *Bb* were similar to the negative control (**Fig 6**).

**Fig 7:**
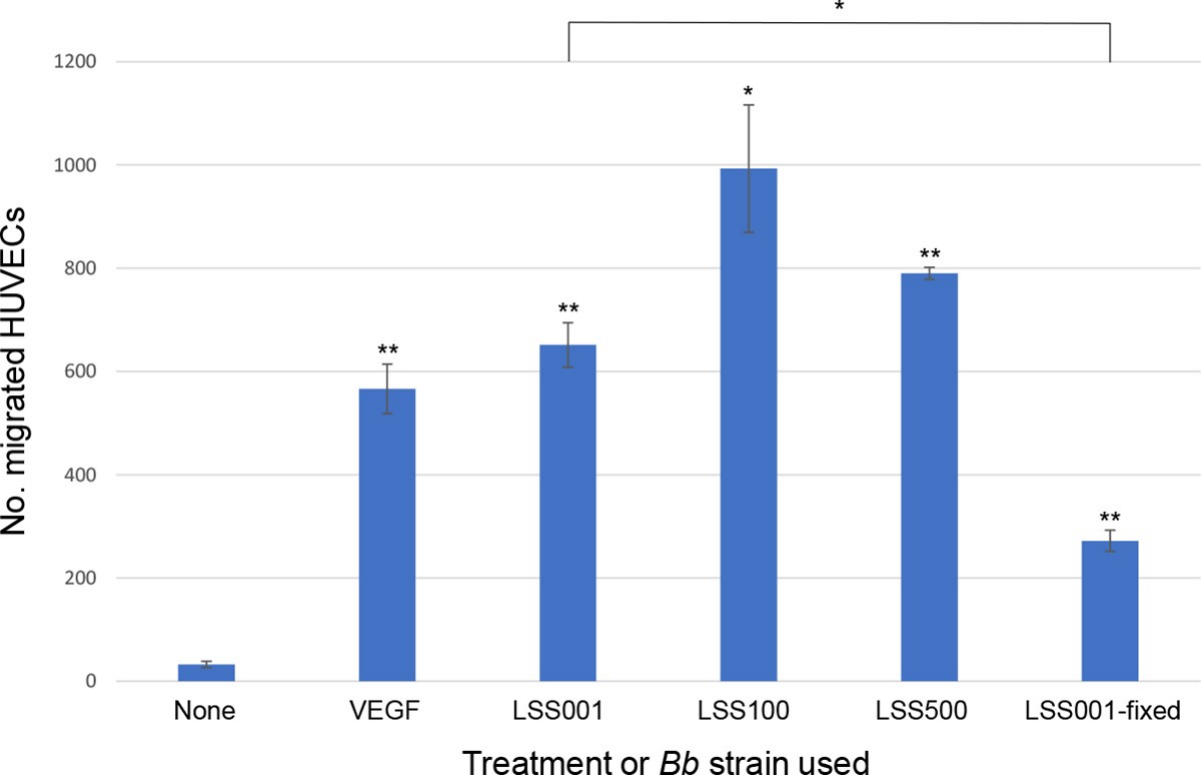
HUVECs migrate towards *Bb* cells. HUVEC migration towards three different live *Bb* strains and non-viable (formaldehyde-fixed) *Bb* was assayed following a 20-h transmigration period. The experiment was conducted independently three times with consistent results. Results of one experiment are shown, and values represent the means of two technical replicates ± SEM. (* *P* < 0.05; ** *P* < 0.01 relative to untreated controls or the indicated datasets).

An early study reported that HUVECs co-cultured with *Bh* were stimulated to migrate in vitro, and the source of the activity was localized to the insoluble fraction of the *Bh* cell [20]. In contrast, a second study showed that *Bb* infection of HUVECs caused intracellular cytoskeletal stress fibers which, in turn, impaired migration [21]. With this apparent discrepancy as a backdrop, we wished to examine the HUVEC migratory response to several *Bb* strains in order to help clarify the host cell’s migratory behavior. To that end, we employed three different live *Bb* strains (LSS001, LSS100 and LSS500) and a non-viable *Bb* strain (fixed LSS001) in an *in vitro* transmigration assay, where the bacteria and host cells were initially separated by a membrane [16]. Results of the assay clearly showed that all *Bb* strains triggered significantly higher HUVEC migration towards the pathogen, as compared to untreated host cells (**Fig 7**). Moreover, migratory activity was comparable to the VEGF-treated positive control cells. To examine whether enhanced migration required viable bacteria as a stimulus, the response to non-viable (formaldehyde-fixed) *Bb* was also assayed. Although fixing *Bb* significantly reduced the activity relative to the corresponding, live *Bb* strain (*P* < 0.05), migratory stimulation by dead bacteria remained significantly higher than for untreated HUVECs (P <0.01). In addition to intact bacteria, we also assayed the HUVEC migration response to cell lysates prepared from five *Bb* strains. These results showed that the migratory activity of HUVECs was significantly stimulated by cell lysates relative to untreated controls and regardless of *Bb* strain (**S3 Fig**).

**Fig 8:**
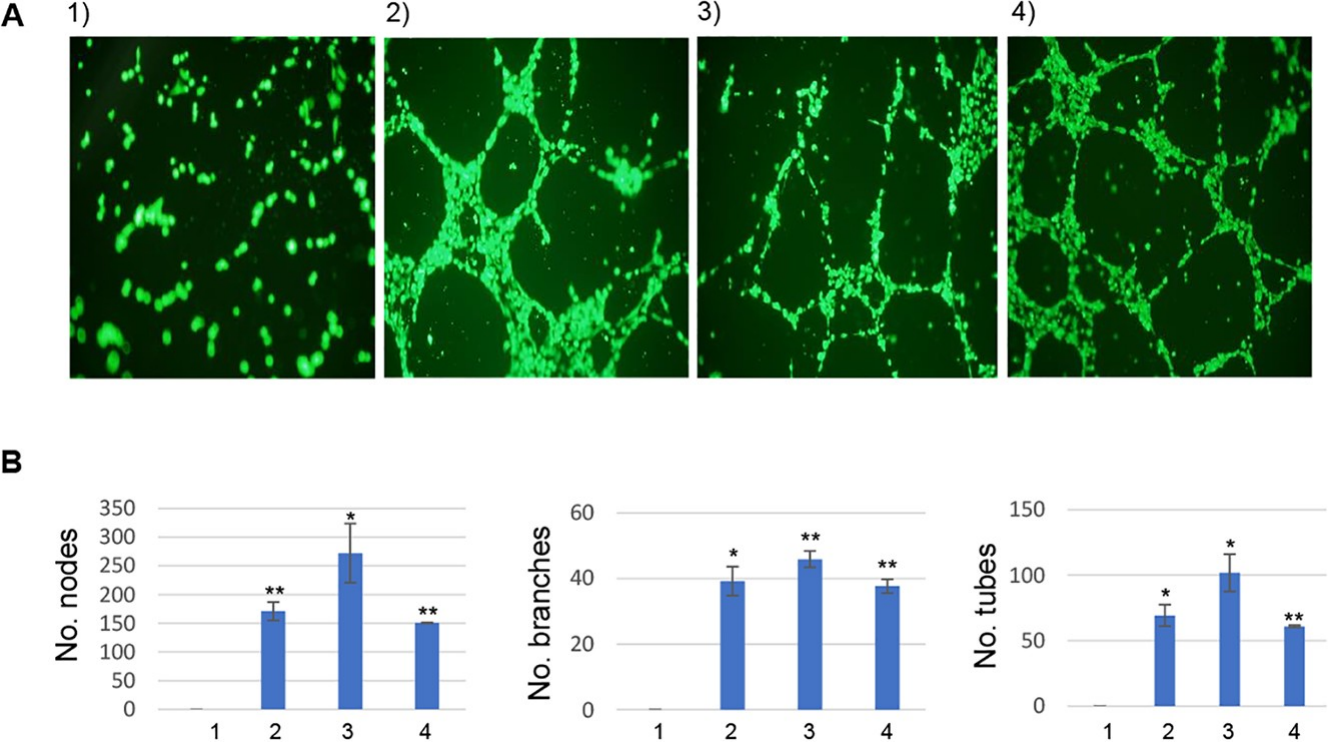
*Bb*-Infected HUVECs form tubes that resemble those induced by VEGF. **A)** Tube formation by HUVECs in response to: 1) untreated culture medium, 2) 6-h VEGF treatment (10 ng/ml), 3) 6-h infection by viable *Bb* LSS100 or 4) exposure to non-viable *Bb* (formaldehyde-fixed LSS100). Examples of typical micrographs (40X magnification) are shown. **B)** Average number of nodes, branches and tubes (master segments and branches) ± SEM in captured micrographs such as those in **Fig 8A**, using the ImageJ Angiogenesis Analyzer plugin [18] is shown. The experiment was done twice independently with five technical replicates each. (* *P* < 0.05; ** *P* < 0.01 relative to untreated controls).

Formation of capillary tube-like structures by stimulated vascular endothelial cells is a hallmark characteristic of angiogenesis, and previous reports have shown that *Bh*-infected HUVECs produce capillary-like tubes in Matrigel [2, 22]. However, a single report involving *Bb* suggested that the intracellular cytoskeletal stress fibers formed during intracellular infection of HUVECs impaired formation of capillary tubes in a collagen matrix [21]. To address this apparent discrepancy, we examined and assayed the formation of nodes, branches and tubes in HUVEC cultures infected with viable or non-viable (formaldehyde-fixed) *Bb* and compared these to VEGF-treated or untreated host cells as positive and negative controls, respectively. Results showed capillary-like tube formation in *Bb*-infected HUVECs that was indistinguishable from VEGF-treated controls (**Fig 8A**). Unexpectedly, tube formation was also observed in HUVECs treated with non-viable *Bb* LSS100. Untreated cells did not produce anastomosing tube-like structures as seen in response to other treatments. Quantification of tube formation by ImageJ analysis of culture images revealed significant increases in the number of nodes, branches and tubes in VEGF-treated and *Bb* infections relative to untreated controls (**Fig 8B**). Differences in the number of nodes, branches and tubes were not significantly different between VEGF-treated HUVECs and *Bb* infections.

We also assayed the HUVEC tube-stimulating activity of cell lysates prepared from five different *Bb* strains. These experiments showed that regardless of *Bb* strain, corresponding cell lysates stimulated HUVECs to form anastomosing tube-like structures that were indistinguishable from VEGF-treated positive controls (**S4A Fig**). As expected, untreated cells did not produce anastomosing tube-like structures. Quantification of tube formation by ImageJ analysis revealed significant increases in the number of nodes, branches and tubes in response to VEGF and *Bb* cell lysates vs. untreated controls, but differences between VEGF-treated cells and the five *Bb* lysate treatments were not significant (**S4B Fig**).

## Discussion

EGF is a 53-amino acid (~6 kDa) polypeptide and a ligand for the EGF receptor (EGFR). EGF plays essential roles in the growth and survival of several cell types through activation of the EGFR signaling pathway and subsequent regulation of cell differentiation, apoptosis, proliferation and migration [23]. To our knowledge, a role for EGF in VEC proliferation during a *Bb* infection has not been previously demonstrated. However, a previous report examining the cytokine profiles of 144 healthy patients from five villages in Northern Peru that had suffered recent *Bb* outbreaks and/or were endemic for Carrión’s disease showed a moderate positive correlation between EGF levels and chronic *Bb* bacteremia [24]. Moreover, the authors suggested that EGF could be involved in the pathological angiogenesis (i.e., verruga peruana) that is commonly observed in the chronic phase of human bartonellosis. Whether the source of elevated levels in these patients was due to infected VECs is unknown but possible in view of our results.

In this study, EGF production by 24-h infected HUVECs was ~5-fold greater than uninfected cells when *Bb* strain LSS001 was used in infection and ~7-fold and significantly greater when *Bb* strains LSS100 or LSS500 were used (*P* < 0.05). These *Bb* strains are isogenic except for plasmid content (**S1 Table**) plus LSS100 and LSS500 overexpress recombinant GroEL (rGroEL) [25]. Taken as a whole, the results suggest that higher levels of rGroEL produced by these two strains fosters increased EGF production by infected HUVECs, perhaps due to enhanced bacterial resistance to intracellular stress through GroEL’s chaperone activities. Interestingly, enhanced EGF production by HUVECs was abrogated if *Bb* was killed by fixing with formaldehyde prior to challenge (**Fig 3**) or if a *Bb* cell lysate was used (**S2 Fig**), suggesting that an active bacterial infection was necessary for increased EGF production.

There are several reports on the molecular basis of hyperproliferation of human VECs in response to a *Bartonella* infection, but the effectors involved are not uniform between species and a demonstrated role for EGF has not been reported, to date. To corroborate the ELISA results identifying human EGF in growth media, we determined that HUVEC proliferation in response to *Bb* infection or VEGF treatment could be blocked by tyrphostin AG1478; a potent and selective EGFR inhibitor (**Fig 4**). From a combination of these two sets of data, we propose that EGF is being produced by *Bb*-infected HUVECs and VEGF-stimulated cells, then subsequently acting to stimulate cell division. Previous work has demonstrated the mitogenic activity of human EGF for HUVECs [26]. As observed with EGF production levels (**Fig 2**), the mitogenic response was significantly greater during infection by LSS100 vs. LSS001, suggesting a possible role for GroEL in HUVEC proliferation, perhaps by enhancing bacterial resistance to intracellular stress.

Conditioned media (CM) containing EGF secreted by *Bb*-infected HUVECs was able to stimulate proliferation of cultured naïve HUVECs in vitro (**Fig 5**). These results suggest that EGF in the CM can act in a paracrine fashion, although it certainly does not rule out the possibility of autocrine activity. Interestingly, blocking CM-grown HUVECs with AG1478, while significantly decreasing cell proliferation, did not abrogate activity to background levels (**Fig 6**), as was observed in AG1478-blocked *Bb* infections (**Fig 4**), suggesting that additional growth factors could be present in the CM.

In addition to discovering a potential role for EGF in *Bb*-induced hyperproliferation of VECs, results of this study also helped to clarify discrepancies regarding the migratory behavior and capillary-like tube formation of HUVECs in response to *Bb*. In the single previous study examining the migratory behavior of *Bb*-infected HUVECs, Verma et al. [21] examined cell migration during infection. In this study, we examined the migratory behavior of HUVECs towards live or dead *Bb* cells (**Fig 7**) or their corresponding cell lysates (**S3 Fig**). While our results suggested that HUVECs migrated towards all *Bb* strains tested, a dead strain evoked a significantly lower migratory response than its live counterpart, but it was still significantly greater than random migration (i.e., untreated controls). One possible explanation for this is that protein cross-linking during formaldehyde treatment only partially inhibited the passive release of a bacterial chemotactic factor(s) from dead bacteria. Interestingly, *Bb* cell lysates uniformly and significantly stimulated migration (**S3 Fig**). It is possible that lysing the *Bb* cell generated a mixture that was rich in a soluble, unidentified chemotactic factor(s) for HUVECs which significantly enhanced migration. In fact, on the whole, the number of HUVECs migrating towards *Bb* cell lysates was higher than the migratory response towards intact bacteria. The role of EGF in HUVEC migration towards *Bb* is unknown but currently under investigation. Capillary-like tube formation was also investigated and found to occur in response to *Bb* infection, challenge by dead *Bb*, or treatment with *Bb* cell lysates. The results showing a lack of EGF production by HUVECs in response to fixed *Bb* cells (**Fig 3**) or *Bb* cell lysates (**S2 Fig)**; both of which evoked tube formation (**Fig 8A** and **S4 Fig**), suggest that the stimulation of capillary-like tube formation can occur independently of EGF production.

The role of EGF in hyperproliferation of host cells during a bacterial infection is not commonly described in the literature. In one study, a link was made between heparin-binding EGF-like growth factor (HB-EGF) and the mucosal epithelial hyperplasia that is commonly observed during otitis media [27]. In another study, levels of EGF, EGFR and a homolog of EGFR (c-erb-B2) were shown to be elevated in gastric mucosa of patients suffering from chronic gastritis with a *Helicobacter pylori* infection, and were comparable to samples from gastric cancer patients [28]. Subsequent work showed that *H*. *pylori* possessing an intact T4SS induced gastrin promoter activity through HB-EGF and EGFR [29]. In turn, gastrin could affect a variety of epithelial cell activities, including proliferation [30].

In summary, we have discovered a potentially novel strategy whereby *Bb* could enhance proliferation and production of additional VEC hosts by inducing synthesis of EGF during infection.

## Supporting information

S1 Fig Map depicting deletion constructs of the *Bb groESL* operon cloned into pGRO1. Intervening blank regions of *groES* (blue) or *groEL* (red) correspond to deleted regions of each gene. Matching plasmid designations and corresponding strains are given to the right. *P* indicates the relative position of the operon’s promoter.
(PPTX)

S2 Fig EGF concentrations (pg/ml) in culture media following a 24-h incubation of HUVECs with cell lysates of the three indicated *Bb* strains, as determined by ELISA. Values represent the means of three independent determinations with two technical replicates each ± SEM. No statistically significant differences were observed between the samples.
(PPTX)

S3 Fig Assays of HUVEC migration over a 20-h period in response towards *Bb* cell lysates (1 μg/ml) prepared from the indicated strains. Experiments were conducted independently three times with consistent results. Results of one experiment are shown, where values represent the means of two technical replicates ± SEM. (* *P* < 0.05; ** *P* < 0.01 relative to untreated controls).
(PPTX)

S4 Fig Tube formation by HUVECs in response to a 6-h treatment with VEGF (10 ng/ml) or cell lysates from the indicated *Bb* strains. Untreated cultures served as controls. **A)** Examples of typical micrographs are shown at 40X magnification. **B)** The number of nodes, branches and tubes in the micrographs is shown, using the ImageJ Angiogenesis Analyzer plugin [18]. Numbers on the X axis correspond to treatments shown in S4A Fig. The experiment was done twice independently with two technical replicates each. (* *P* < 0.05; ** *P* < 0.01 relative to untreated controls).
(PPTX)

S1 Table Bacterial strains and plasmids used in the study. (DOCX)

S2 Table Primers used in the study. (DOCX)

## References

[1] Minnick MF, Anderson BE. *Bartonella*. Chapter 105 In: Tang Y-W, Sussman M, Liu D, Poxton I, Schwartzman J, editors. Molecular Medical Microbiology, Second Edition London: Academic Press; 2014 pp. 1911–1939.

[2] McCord AM, Resto-Ruiz SI, Anderson BE. An autocrine role for IL-8 in *Bartonella henselae*-induced angiogenesis. Infect Immun. 2006; 74:5185–5190. 10.1128/IAI.00622-06 16926411 PMC1594831

[3] McCord AM, Burgess AW, Whaley MJ, Anderson BE. Interaction of *Bartonella henselae* with endothelial cells promotes monocyte/macrophage chemoattractant protein 1 gene expression and protein production and triggers monocyte migration. Infect Immun. 2005; 73:5735–5742. 10.1128/IAI.73.9.5735-5742.2005 16113290 PMC1231114

[4] Resto-Ruiz SI, Schmiederer M, Sweger D, Newton C, Klein TW, Friedman H, et al Induction of a potential paracrine angiogenic loop between human THP-1 macrophages and human microvascular endothelial cells during *Bartonella henselae* infection. Infect Immun. 2002; 70:4564–4570. 10.1128/IAI.70.8.4564-4570.2002 12117969 PMC128175

[5] Kempf VA, Volkmann B, Schaller M, Sander CA, Alitalo K, Riess T, et al Evidence of a leading role for VEGF in *Bartonella henselae*-induced endothelial cell proliferations. Cell Microbiol. 2001; 3:623–632. 10.1046/j.1462-5822.2001.00144.x 11553014

[6] Kempf VA, Lebiedziejewski M, Alitalo K, Wälzlein JH, Ehehalt U, Fiebig J, et al Activation of hypoxia-inducible factor-1 in bacillary angiomatosis: evidence for a role of hypoxia-inducible factor-1 in bacterial infections. Circulation. 2005; 111(8):1054–1062. 10.1161/01.CIR.0000155608.07691.B7 15723970

[7] Schmid MC, Schulein R, Dehio M, Denecker G, Carena I, Dehio C. The VirB type IV secretion system of *Bartonella henselae* mediates invasion, proinflammatory activation and antiapoptotic protection of endothelial cells. Mol Microbiol 2004; 52:81–92. 10.1111/j.1365-2958.2003.03964.x 15049812

[8] Schulein R, Guye P, Rhomberg TA, Schmid MC, Schröder G, Vergunst AC, et al A bipartite signal mediates the transfer of type IV secretion substrates of *Bartonella henselae* into human cells. Proc Natl Acad Sci USA 2005; 102:856–861. 10.1073/pnas.0406796102 15642951 PMC545523

[9] Schmid MC, Scheidegger F, Dehio M, Balmelle-Devaux N, Schulein R, Guye P, et al A translocated bacterial protein protects vascular endothelial cells from apoptosis. PLoS Pathog. 2006; 2(11):e115 10.1371/journal.ppat.0020115 17121462 PMC1657063

[10] Riess T, Andersson SG, Lupas A, Schaller M, Schäfer A, Kyme P, et al *Bartonella* adhesin a mediates a proangiogenic host cell response. J Exp Med. 2004; 200(10):1267–1278. 10.1084/jem.20040500 15534369 PMC2211922

[11] Minnick MF, Anderson BE, Lima A, Battisti JM, Lawyer PG, Birtles RJ. Oroya fever and verruga peruana: bartonelloses unique to South America. PLoS Negl Trop Dis. 2014; 8(7):e2919 10.1371/journal.pntd.0002919 25032975 PMC4102455

[12] Garcia FU, Wojta J, Broadley KN, Davidson JM, Hoover RL. *Bartonella bacilliformis* stimulates endothelial cells in vitro and is angiogenic in vivo. Am J Pathol. 1990; 136(5):1125–1135. 1693472 PMC1877437

[13] Minnick MF, Smitherman LS, Samuels DS. Mitogenic effect of *Bartonella bacilliformis* on human vascular endothelial cells and involvement of GroEL. Infect Immun. 2003; 71(12):6933–6942. 10.1128/IAI.71.12.6933-6942.2003 14638782 PMC308913

[14] Battisti JM, Minnick MF. Development of a system for genetic manipulation of *Bartonella bacilliformis*. Appl Environ Microbiol. 1999; 65(8):3441–3448. 10427032 10.1128/aem.65.8.3441-3448.1999 PMC91517

[15] Connolly DT, Knight MB, Harakas NK, Wittwer AJ, Feder J. Determination of the number of endothelial cells in culture using an acid phosphatase assay. Anal Biochem. 1986; 152:136–140. 10.1016/0003-2697(86)90131-4 3954035

[16] Sherman H, Pardo P, Upton T. Cell migration, chemotaxis and invasion assay protocol. https://www.corning.com/worldwide/en/search.html?productsSearchState=&resourcesSearchState=&relatedContentSearchState=&initialResultType=resources&searchText=cell%20migration%20assay&search-initialcatalog=Corporate%20Communications

[17] Arnaoutova I, Kleinman H. In vitro angiogenesis: endothelial cell tube formation on gelled basement membrane extract. Nat Protoc. 2010; 4:628–635. 10.1038/nprot.2010.6 20224563

[18] Schneider CA, Rasband WS, Eliceiri KW. NIH Image to ImageJ: 25 years of image analysis. Nat Methods. 2012; 9(7):671–675. 10.1038/nmeth.2089 22930834 PMC5554542

[19] Arteaga CL, Ramsey TT, Shawver LK, Guyer CA. Unliganded epidermal growth factor receptor dimerization induced by direct interaction of quinazolines with the ATP binding site. J Biol Chem. 1997 9 12;272(37):23247–23254. 10.1074/jbc.272.37.23247 9287333

[20] Conley T, Slater L, Hamilton K. *Rochalimaea* species stimulate human endothelial cell proliferation and migration in vitro. J Lab Clin Med. 1994 10;124(4):521–528. 7523553

[21] Verma A, Davis GE, Ihler GM. Formation of stress fibres in human endothelial cells infected with *Bartonella bacilliformis* is associated with altered morphology, impaired migration and defects in cell morphogenesis. Cell Microbiol. 2001 3;3(3):169–180. 10.1046/j.1462-5822.2001.00104.x 11260140

[22] Berrich M, Kieda C, Grillon C, Monteil M, Lamerant N, Gavard J, Boulouis HJ, Haddad N. Differential effects of *Bartonella henselae* on human and feline macro- and micro-vascular endothelial cells. PLoS One. 2011;6(5):e20204 10.1371/journal.pone.0020204 21637717 PMC3103534

[23] Citri A, Yarden Y. EGF-ERBB signalling: towards the systems level. Nat Rev Mol Cell Biol. 2006 7;7(7):505–516. 10.1038/nrm1962 16829981

[24] Pons MJ, Gomes C, Aguilar R, Barrios D, Aguilar-Luis MA, Ruiz J, Dobaño C, Del Valle-Mendoza J, Moncunill G. Immunosuppressive and angiogenic cytokine profile associated with *Bartonella bacilliformis i*nfection in post-outbreak and endemic areas of Carrion's disease in Peru. PLoS Negl Trop Dis. 2017 6 19;11(6):e0005684 10.1371/journal.pntd.0005684 28628613 PMC5491314

[25] Smitherman LS, Minnick MF. *Bartonella bacilliformis* GroEL: effect on growth of human vascular endothelial cells in infected cocultures. Ann N Y Acad Sci. 2005 12;1063:286–298. 10.1196/annals.1355.046 16481529 PMC1817666

[26] Hastie R, Tong S, Hannan NJ, Brownfoot F, Cannon P, Kaitu'u-Lino TJ. Epidermal growth factor rescues endothelial dysfunction in primary human tissues in vitro. Reprod Sci. 2017 9;24(9):1245–1252. 10.1177/1933719116681516 27920343

[27] Suzukawa K, Tomlin J, Pak K, Chavez E, Kurabi A, Baird A, Wasserman SI, Ryan AF. A mouse model of otitis media identifies HB-EGF as a mediator of inflammation-induced mucosal proliferation. PLoS One. 2014 7 17;9(7):e102739 10.1371/journal.pone.0102739 25033458 PMC4102546

[28] Jurkowska G, Piotrowska-Staworko G, Guzińska-Ustymowicz K, Kemona A, Świdnicka-Siergiejko A, Łaszewicz W, Dąbrowski A. The impact of *Helicobacter pylori* on EGF, EGF receptor, and the c-erb-B2 expression. Adv Med Sci. 2014 9;59(2):221–226. 10.1016/j.advms.2014.01.006 25051417

[29] Gunawardhana Niluka, Jang Sungil, Yun Hui Choi Youngmin A. Hong, Jeon Yeong-Eui, Kim Aeryun, Su Hanfu, Kim Ji-Hye, Yoo Yun-Jung, Merrell D. Scott, Kim Jinmoon, Cha Jeong-Heon. *Helicobacter pylori*-induced HB-EGF upregulates gastrin expression via the EGF receptor, C-Raf, Mek1, and Erk2 in the MAPK pathway. Front Cell Infect Microbiol. 2017; 7: 541 10.3389/fcimb.2017.00541 29379775 PMC5775237

[30] Dockray GJ. Topical review. Gastrin and gastric epithelial physiology. J Physiol. 1999 7 15;518 (Pt 2):315–324. 10381581 10.1111/j.1469-7793.1999.0315p.x PMC2269421

