# Supplementary figures and images for: Human vascular endothelial cells express epithelial growth factor in response to infection by Bartonella bacilliformis
Source: PLoS Negl Trop Dis. 2020 Apr 17;14(4):e0008236. doi: 10.1371/journal.pntd.0008236 (PMC7190185; doi:10.1371/journal.pntd.0008236)

## Slide 1
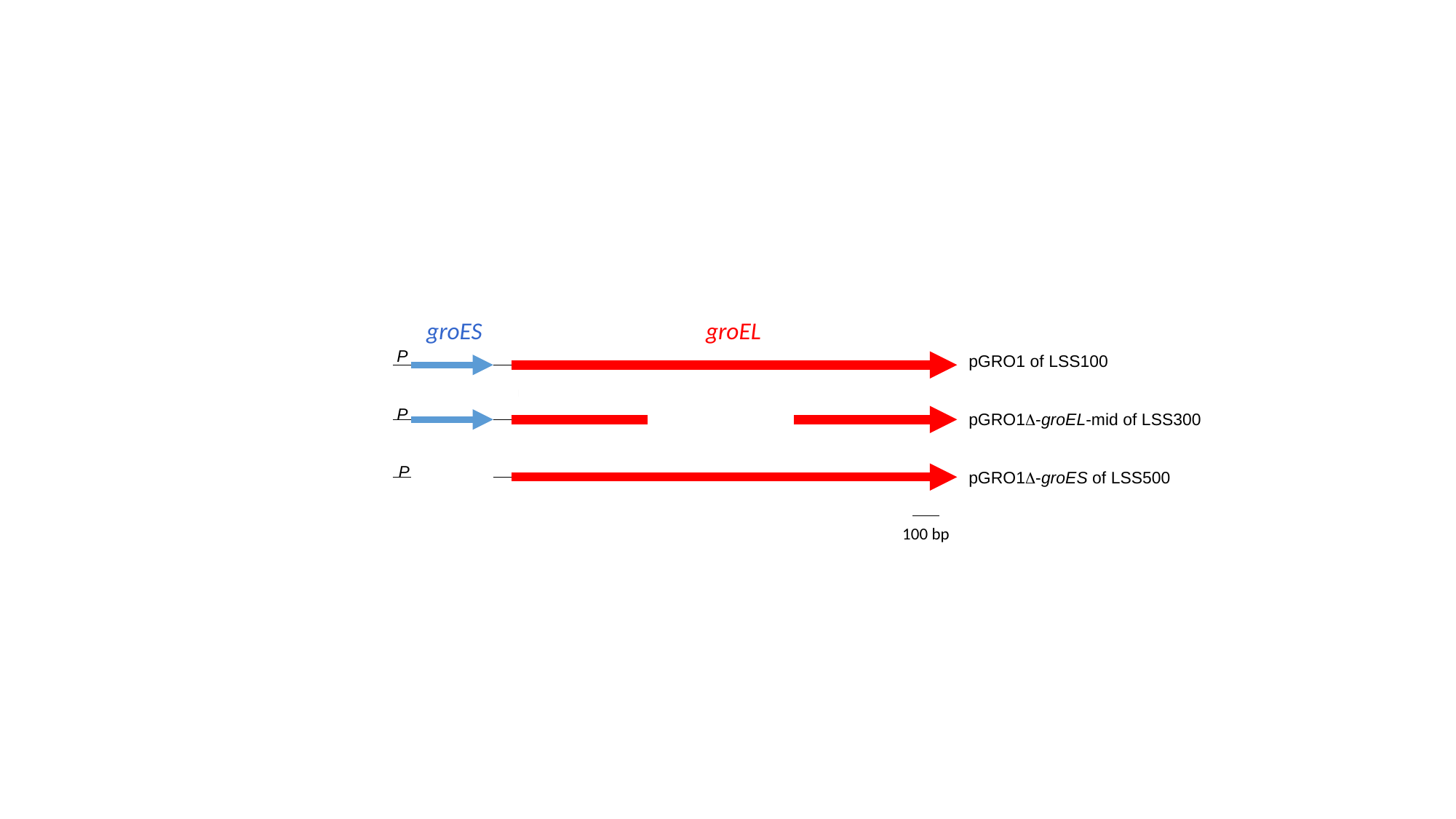

groES groEL
pGRO1 of LSS100
pGRO1D-groEL-mid of LSS300
pGRO1D-groES of LSS500
 100 bp
P
P
P

Supplement: S1 Fig — Intervening blank regions of groES (blue) or groEL (red) correspond to deleted regions of each gene. Matching plasmid designations and corresponding strains are given to the right. P indicates the relative position of the operon’s promoter. (PPTX) [file pntd.0008236.s001.pptx]
