# Supplementary material for: Human vascular endothelial cells express epithelial growth factor in response to infection by Bartonella bacilliformis
Source: PLoS Negl Trop Dis. 2020 Apr 17;14(4):e0008236. doi: 10.1371/journal.pntd.0008236 (PMC7190185; doi:10.1371/journal.pntd.0008236)
Supplement: S2 Fig — Values represent the means of three independent determinations with two technical replicates each ± SEM. No statistically significant differences were observed between the samples. (PPTX) [file pntd.0008236.s002.pptx]

## Slide 1
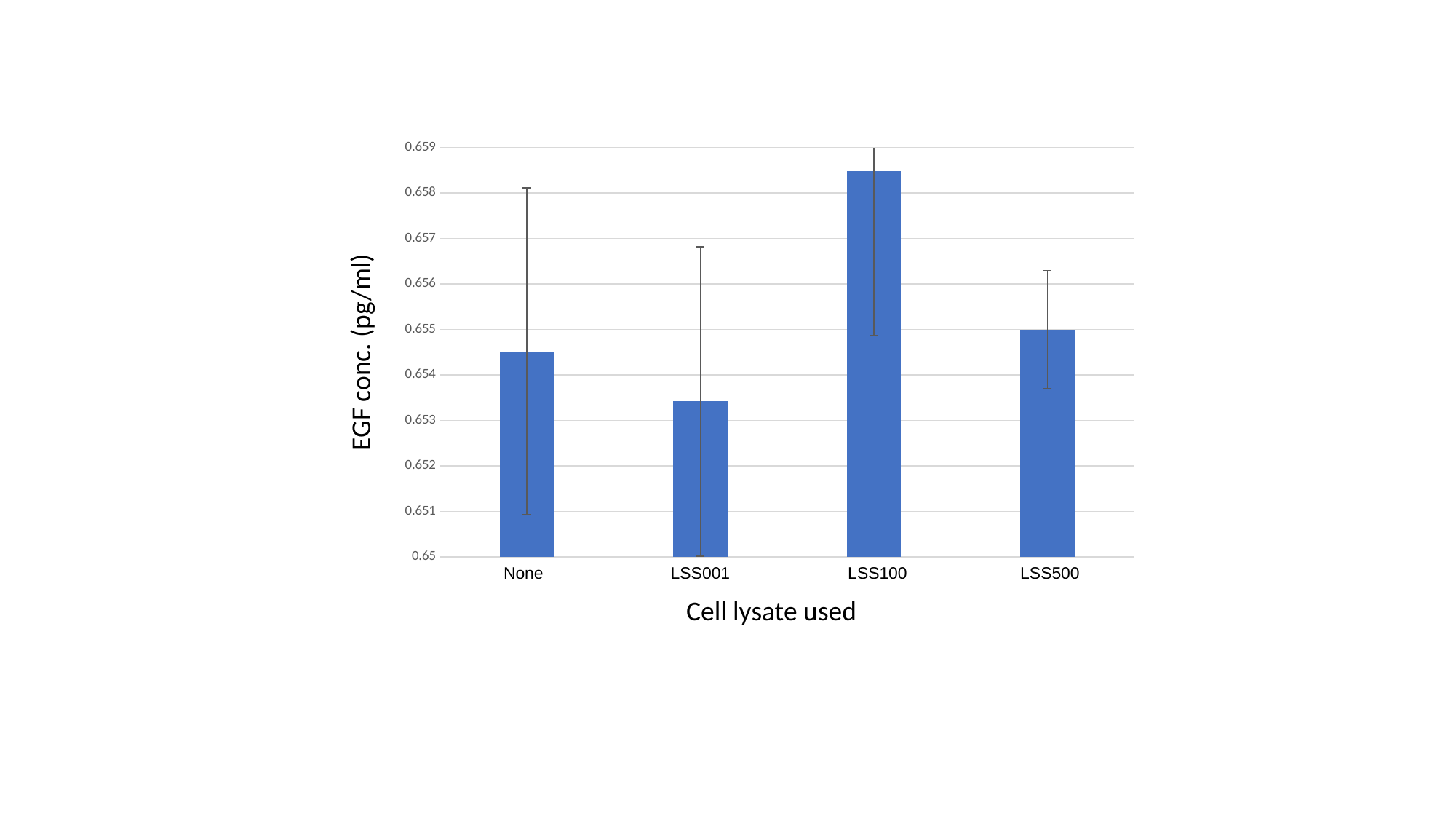

### Chart
| Category | |
|---|---|
| HUVEC | 0.654517439598208 |
| PCB pBBR1MCS2 | 0.6534149904442105 |
| PCB pBBR1MCS2 GroESL | 0.658473361711745 |
| PCB pBBR1MCS2 GroES(-) | 0.6549979917187947 |EGF conc. (pg/ml)
 None LSS001 LSS100 LSS500
Cell lysate used
