# Supplementary material for: Human vascular endothelial cells express epithelial growth factor in response to infection by Bartonella bacilliformis
Source: PLoS Negl Trop Dis. 2020 Apr 17;14(4):e0008236. doi: 10.1371/journal.pntd.0008236 (PMC7190185; doi:10.1371/journal.pntd.0008236)
Supplement: S3 Fig — Experiments were conducted independently three times with consistent results. Results of one experiment are shown, where values represent the means of two technical replicates ± SEM. (* P < 0.05; ** P < 0.01 relative to untreated controls). (PPTX) [file pntd.0008236.s003.pptx]

## Slide 1
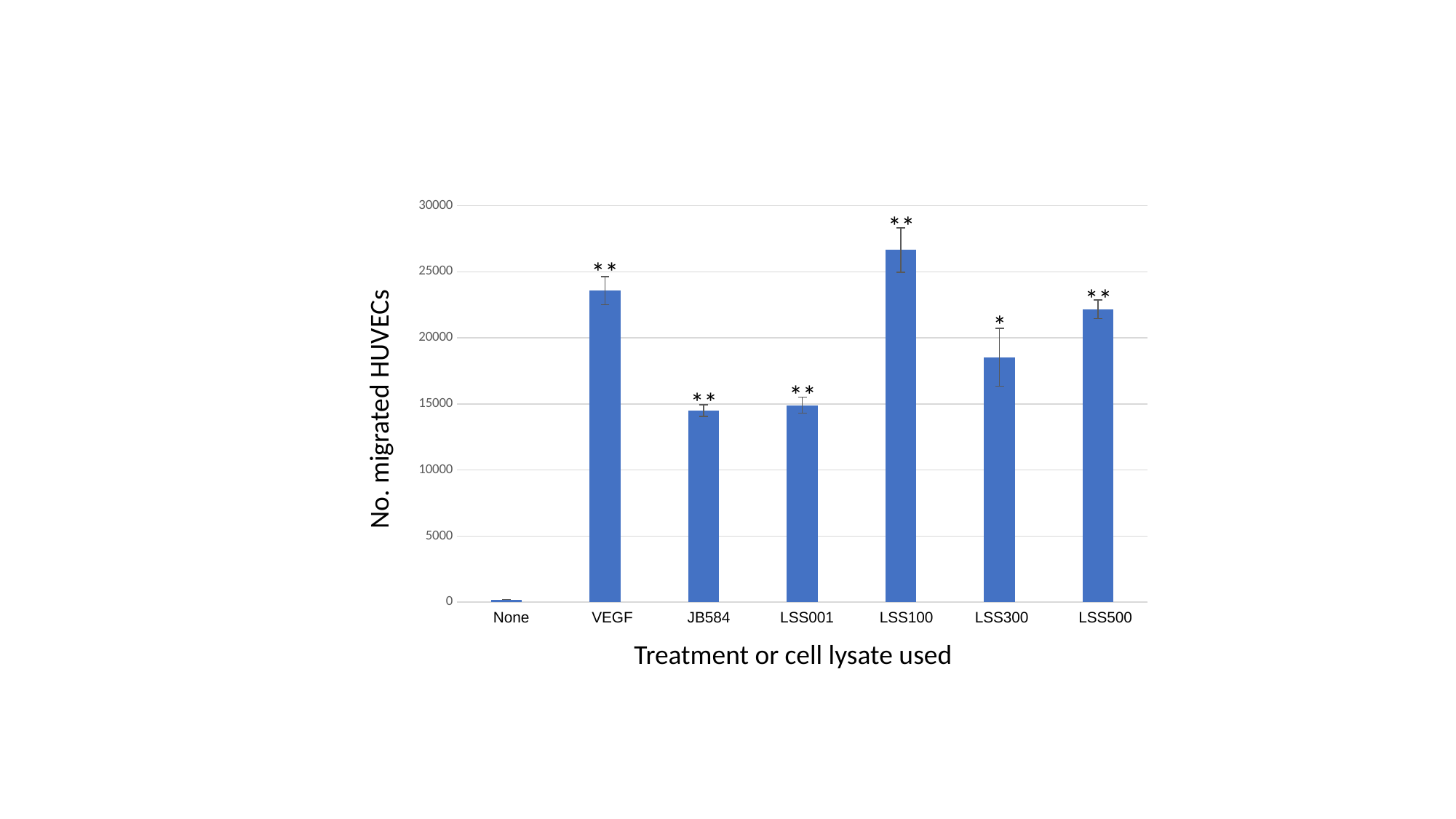

### Chart
| Category | |
|---|---|
| 0%FCS | 198.0 |
| VEGF | 23573.0 |
| PCB 1ug/ml | 14498.0 |
| pBBR 1ug/ml | 14905.0 |
| GroESL 1ug/ml | 26642.0 |
| D1 1ug/ml | 18535.0 |
| GroES(-) 1ug/ml | 22154.0 |**
**
**
*
**
**
No. migrated HUVECs
 None VEGF JB584 LSS001 LSS100 LSS300 LSS500
Treatment or cell lysate used
