# Supplementary material for: Human vascular endothelial cells express epithelial growth factor in response to infection by Bartonella bacilliformis
Source: PLoS Negl Trop Dis. 2020 Apr 17;14(4):e0008236. doi: 10.1371/journal.pntd.0008236 (PMC7190185; doi:10.1371/journal.pntd.0008236)
Supplement: S4 Fig — Untreated cultures served as controls. A) Examples of typical micrographs are shown at 40X magnification. B) The number of nodes, branches and tubes in the micrographs is shown, using the ImageJ Angiogenesis Analyzer plugin [18]. Numbers on the X axis correspond to treatments shown in S4A Fig. The experiment was done twice independently with two technical replicates each. (* P < 0.05; ** P < 0.01 relative to untreated controls). (PPTX) [file pntd.0008236.s004.pptx]

## Slide 1
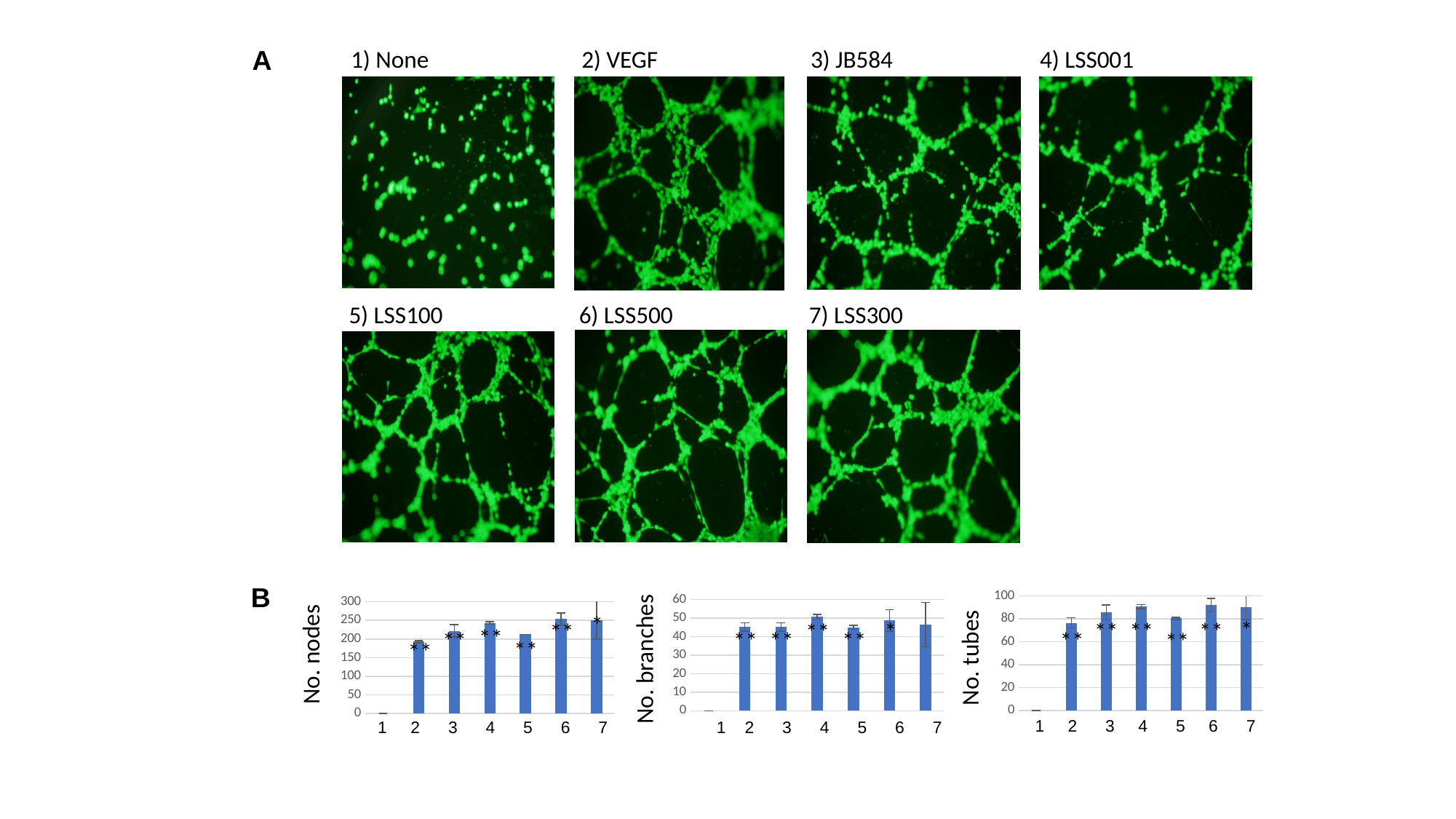

A
 1) None 2) VEGF 3) JB584 4) LSS001
5) LSS100 6) LSS500 7) LSS300
B
### Chart
| Category | |
|---|---|
| No FCS | 0.0 |
| VEGF | 76.5 |
| PCB | 85.5 |
| PCB pBBR1MCS2 | 90.75 |
| PCB pBBR1MCS2 GroESL | 80.5 |
| PCB pBBR1MCS2 GroES (minus) | 92.0 |
| PCB pBBR1MCS2 GroESL D1 | 90.25 |
### Chart
| Category | Avg # Nodes |
|---|---|
| No FCS | 0.0 |
| VEGF | 192.75 |
| PCB | 221.75 |
| PCB pBBR1MCS2 | 243.0 |
| PCB pBBR1MCS2 GroESL | 212.5 |
| PCB pBBR1MCS2 GroES (minus) | 253.5 |
| PCB pBBR1MCS2 GroESL D1 | 250.0 |
### Chart
| Category | Avg # Branches |
|---|---|
| No FCS | 0.0 |
| VEGF | 45.25 |
| PCB | 45.25 |
| PCB pBBR1MCS2 | 50.75 |
| PCB pBBR1MCS2 GroESL | 45.0 |
| PCB pBBR1MCS2 GroES (minus) | 48.75 |
| PCB pBBR1MCS2 GroESL D1 | 46.5 |*
*
**
**
*
**
**
**
**
**
**
**
**
**
**
**
**
No. nodes
No. tubes
No. branches
 1 2 3 4 5 6 7
 1 2 3 4 5 6 7
 1 2 3 4 5 6 7
