# Supplementary material for: Human vascular endothelial cells express epithelial growth factor in response to infection by Bartonella bacilliformis
Source: PLoS Negl Trop Dis. 2020 Apr 17;14(4):e0008236. doi: 10.1371/journal.pntd.0008236 (PMC7190185; doi:10.1371/journal.pntd.0008236)
Supplement: S1 Table — (DOCX) [file pntd.0008236.s005.docx]

**S1 Table**

**Bacterial strains and plasmids used in the study.**

**Strain or plasmid Relevant characteristic / phenotype Source or reference**

***B. bacilliformis***

JB584 Transformable strain of *B. bacilliformis* [1]

LSS001 JB584 containing pBBR1MCS2. Control [2]

strain with shuttle vector only.

LSS100 JB584 containing pGRO1. Overexpresses [2]

rGroES and rGroEL.

LSS300 JB584 containing pGRO1Δ−*groEL*-mid. This study

Overexpresses rGroES but not rGroEL.

LSS500 JB584 containing pGRO1Δ−*groES* This study

Overexpresses rGroEL but not rGroES.

***E. coli***

TOP10F' Host strain for genetic manipulation Invitrogen

and plasmid production

**Plasmid**

pBBR1MCS2 Broad host-range shuttle vector; Kan^r^ [3]

pGRO1 pBBR1MCS2 with cloned *B.* [2]

*bacilliformis groESL* operon

pGRO1Δ−*groEL*-mid pGRO1 with ~1400-bp deletion in This study

mid-section of *groEL* gene

pGRO1Δ−*groES* pGRO1 with ~300-bp deletion to This study

delete *groES* and retain promoter

**Bibliography-**

# [1]. [Battisti JM](https://www.ncbi.nlm.nih.gov/pubmed/?term=Battisti%20JM%5BAuthor%5D&cauthor=true&cauthor_uid=10427032), [Minnick MF](https://www.ncbi.nlm.nih.gov/pubmed/?term=Minnick%20MF%5BAuthor%5D&cauthor=true&cauthor_uid=10427032). Development of a system for genetic manipulation of *Bartonella* *bacilliformis*. [Appl Environ Microbiol.](https://www.ncbi.nlm.nih.gov/pubmed/?term=battisti%2C+minnick+1999) 1999; 65(8):3441-3448.

# [2]. Minnick MF, Smitherman LS, Samuels DS. [Mitogenic effect of *Bartonella bacilliformis* on human vascular endothelial cells and involvement of GroEL.](https://www.ncbi.nlm.nih.gov/pubmed/14638782) Infect Immun. 2003; 71(12):6933-42.

[3]. Kovach ME, Elzer PH, Hill DS, Robertson GT, Farris MA, Roop RM 2nd, et al. [Four new derivatives of the broad-host-range cloning vector pBBR1MCS, carrying different antibiotic-resistance cassettes.](https://www.ncbi.nlm.nih.gov/pubmed/8529885) Gene. 1995; 166(1):175-6.
