# Supplementary material for: Human vascular endothelial cells express epithelial growth factor in response to infection by Bartonella bacilliformis
Source: PLoS Negl Trop Dis. 2020 Apr 17;14(4):e0008236. doi: 10.1371/journal.pntd.0008236 (PMC7190185; doi:10.1371/journal.pntd.0008236)
Supplement: S2 Table — (DOCX) [file pntd.0008236.s006.docx]

**S2 Table**

**Primers used in the study.**

**Plasmid construct Primer designation Primer sequence**

pBBR1-MCS2*^a^* pbbrSEQ_F  CAGGGTTTTCCCAGTCAC

pbbrSEQ_R  TTAGGCACCCCAGGCTTTACAC

pGRO1Δ−*groEL*-mid*^b^* Q5GroESL_D1_F AAATTGCAAGAAAGACTTG

Q5GroESL_D1_R TTTTTGCTTCTTCTACCG

pGRO1Δ−*groES^b^* Q5GroESDelta_F ATCCGATTTTCCATTATTTG

Q5GroESDelta_R GTTTTAAACCCTTAAAACTTG

*^a^*For automated sequencing of pBBR1-MCS2 constructs

*^b^*For generating deletion mutations of the *Bb groESL* operon of pGRO1 using a Q5 Site-Directed Mutagenesis kit (New England Biolabs).
